# A Feasibility Study of High-Entropy Alloy Coating Deposition by Detonation Spraying Combined with Laser Melting

**DOI:** 10.3390/ma15134532

**Published:** 2022-06-27

**Authors:** Igor S. Batraev, Vladimir Yu. Ulianitsky, Alexey A. Sova, Marina N. Samodurova, Evgeny A. Trofimov, Kirill Yu. Pashkeev, Alexander G. Malikov, Dina V. Dudina, Arina V. Ukhina

**Affiliations:** 1 Lavrentyev Institute of Hydrodynamics SB RAS, 630090 Novosibirsk, Russia; ibatraev@gmail.com (I.S.B.); ulianv@mail.ru (V.Y.U.); 2 ECL-ENISE, LTDS Laboratory UMR 5513, University of Lyon, 42000 Saint-Etienne, France; sova.aleksey@gmail.com; 3 Department of Information and Measuring Technology, South Ural State University, 454080 Chelyabinsk, Russia; samodurovamn@susu.ru (M.N.S.); tea7510@gmail.com (E.A.T.); kjpashkeev@gmail.com (K.Y.P.); 4 Khristianovich Institute of Theoretical and Applied Mechanics SB RAS, 630090 Novosibirsk, Russia; smalik@ngs.ru; 5 Institute of Solid State Chemistry and Mechanochemistry SB RAS, 630090 Novosibirsk, Russia; auhina181@gmail.com

**Keywords:** detonation spraying, high-entropy alloy, composite coating, laser melting

## Abstract

In this work, a new two-stage approach to the deposition of high-entropy alloy coatings is proposed. At the first stage, a composite precursor coating is formed by detonation spraying of the metal powder mixtures. At the second stage, the precursor coating is re-melted by a laser, and the formation of multi-component solid solution phases can be expected upon solidification. The feasibility of the proposed approach was validated using three different mixtures of Fe, Ni, Cu, Co and Al powders. It was shown that detonation spraying allows forming composite coatings with a uniform distribution of the lamellae of different metals. The results of the structural analysis of the laser-treated coatings suggest that complete alloying occurred in the melt and face-centered cubic solid solutions formed in the coatings upon cooling.

## 1. Introduction

The fabrication of high-entropy alloy (HEA) coatings is an emerging direction in the development of thermal spray technologies. HEAs possess a high application potential in mechanical, chemical, aerospace and automotive industries [1]. Due to the high value of the mixing entropy of a solid solution, HEAs are more stable than the corresponding intermetallic compounds, especially at high temperatures. HEAs are defined as alloys containing several principal elements, each having an atomic percentage between 5% and 35% [2]. However, some authors suggest that only alloys that form a solid solution and do not contain intermetallic phases should be considered as true HEAs [1].

**Figure 1:**
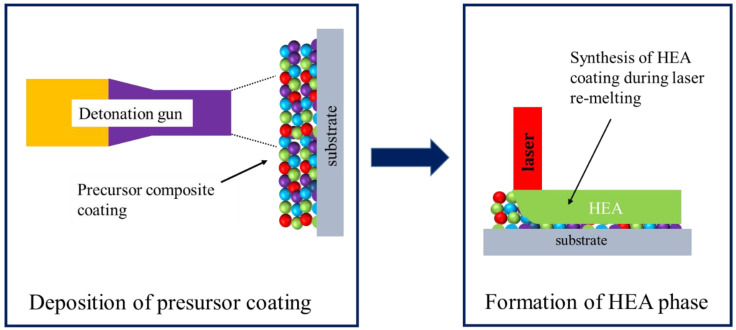
Illustration of the two-stage approach to the formation of HEA coatings: deposition of a precursor coating by detonation spraying followed by laser re-melting.

The successful deposition of HEA coatings by high-velocity oxy-fuel spraying [3,4], atmospheric plasma spraying [5,6] and detonation spraying [7] has been reported. It is important to note that, in all those works, the HEA coatings were thermally sprayed using pre-alloyed powders produced by ball milling or gas atomization. It should be noted that manufacturing of HEA powders, especially those with customized compositions, is an expensive and complicated process. Currently, only a limited number of HEA feedstock powders are available on the market. In this regard, we propose a new two-stage approach to the deposition of HEA coatings. The basic principles of this hybrid approach are illustrated in Figure 1. At the first stage, a precursor composite coating consisting of metallic components is deposited by detonation spraying. The elemental composition of the coating should be close to the target composition of the HEA. It is clear that the composite coating itself cannot be considered as a true HEA coating, as only a limited chemical interaction between the deposited particles occurs during the coating formation. In order to promote the formation of solid solutions in the coating, post-treatment should be applied at the second stage. In particular, the coating can be treated by a laser beam to induce melting of the coating components. The coating will be transformed into a liquid and solid solutions will be formed upon solidification of the melt.

It is important to note that, in the case of successful development, this hybrid approach would allow depositing HEA coatings with a wide range of elemental compositions. Most metals can be deposited by detonation spraying [8,9,10]. Previous studies showed that metal composite coatings containing three, four or five components can be deposited successfully by detonation spraying [11]. A proper adjustment of the precursor coating composition and optimization of the post-treatment procedure could potentially allow fabricating HEA coatings with almost any combination of metallic elements.

The purpose of this work was to perform a feasibility test of the proposed two-stage approach and answer the following questions:

- Is there a difference between the composition of the powder mixture and the coating formed by detonation spraying?
- Will the generation of a molten pool by the laser treatment enable the formation of a solid solution in the re-solidified coating?
- The temperature gradients and heating/cooling rates during laser melting are very high. Are these thermal conditions suitable for the formation of dense coatings containing solid solution phases?

## 2. Materials and Methods

**Figure 2:**
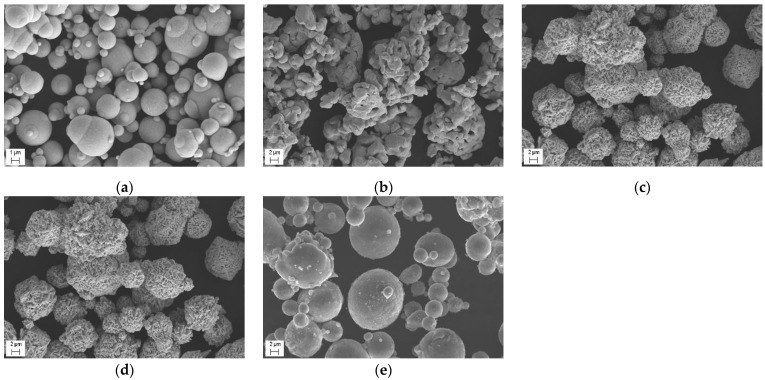
Scanning electron microscopy (SEM) images of the powders used for the experiments: (**a**) iron; (**b**) cobalt; (**c**) nickel; (**d**) copper; (**e**) aluminum.

The precursor coatings were deposited using five metal powders (cobalt, nickel, iron, copper and aluminum) as the starting materials. The morphology of the powders is seen in the images presented in Figure 2. The measurements of the particle size distribution showed that the mean particle sizes of the powders are close to each other (Table 1).

In order to find the compositions that could potentially be suitable for the formation of multi-component solid solution structures, the thermodynamic calculations were carried out. At present, different thermodynamic, electrochemical and topological criteria are applied to predict the solid solution formation in multi-component alloys [1,12,13]. The parameters most widely used for these predictions are the enthalpy of mixing, *ΔH_mix_*, the entropy of mixing, *ΔS_mix_*, and the atomic size difference, *δ*. Zhang et al. [12] found that single solid solutions form in alloys with parameters satisfying the criteria −20 < *ΔH_mix_* < 5 kJ/mol, 12 < *ΔS_mix_* < 17.5 J/K mol, and *δ* < 6.4%. Guo and Liu [13] stated that a solid solution is expected to form if all three parameters meet the following requirements: −22 ≤ *ΔH_mix_* ≤ 7 kJ/mol, 11 ≤ *ΔS_mix_* ≤ 19.5 J/K mol, and 0 ≤ *δ* ≤ 8.5.

Experiments were conducted on three different alloy compositions. In order to select the compositions, the mixing enthalpy, mixing entropy and atomic size difference were calculated. The values of the mixing enthalpies of the atomic pairs used for the calculations are given in Table 2. The calculations allowed us to determine the composition of the mixtures (Table 3). Mixture 1 (designated as Mix 1) perfectly matches the topological and mixing enthalpy criteria for the formation of a single-phase solid solution. The mixing enthalpy, mixing entropy and atomic size mismatch of mixture 3 (Mix 3) are in the required ranges. The mixing entropy and the atomic size mismatch of mixture 2 (Mix 2) satisfy the requirements, but its mixing enthalpy is higher than the boundary of the range defined by Guo and Liu (9.99 kJ/mol vs. 7 kJ/mol).

**Figure 3:**
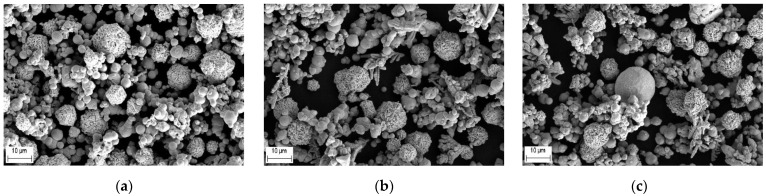
SEM images of the powder mixtures used for the deposition of the precursor coatings: (**a**) Mix 1; (**b**) Mix 2; (**c**) Mix 3.

The mixtures were prepared using a laboratory mixer. The mixing time was 30 min. The SEM images of the mixtures are presented in Figure 3.

**Figure 4:**
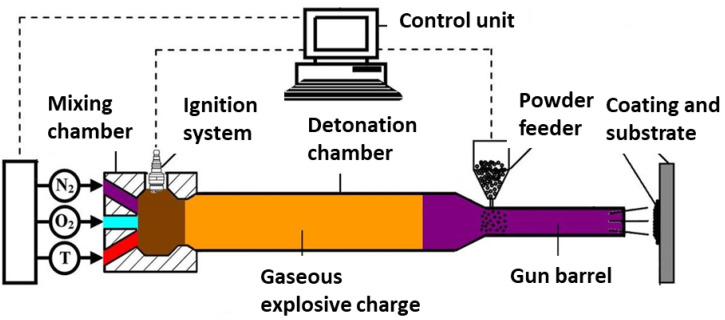
Schematic of a CCDS2000 detonation gun.

The mixtures were deposited on mild steel substrates with a thickness of 3 mm. The substrates were sandblasted prior to deposition. The detonation spraying experiments were carried out using a CCDS2000 detonation spraying facility developed at Lavrentyev Institute of Hydrodynamics SB RAS (Novosibirsk, Russia). A schematic of the detonation gun is presented in Figure 4. A double-section barrel with a conical transition was used. The detonation chamber had a length of 650 mm and a diameter of 26 mm. The muzzle section had a length of 300 mm and a diameter of 20 mm. The injection point of the powder was located at a distance of 300 mm from the barrel exit. The distance between the barrel exit and the substrate was 100 mm.

**Figure 5:**
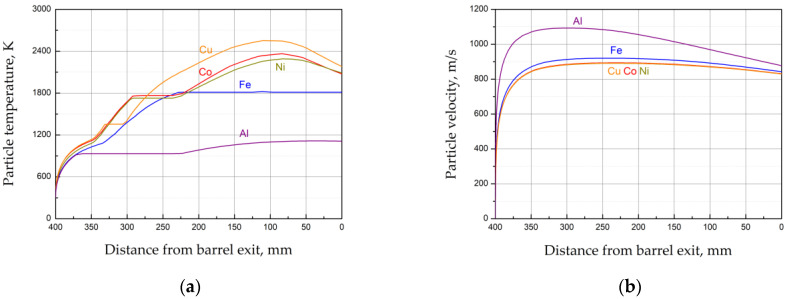
The calculated temperatures (**a**) and velocities (**b**) of the particles of the metals. Calculations for particles 10 μm in size.

In order to prevent the oxidation of the powders during spraying, an equimolar acetylene–oxygen mixture (C_2_H_2_ + O_2_) was used as the explosive charge. The dilution of the explosive charge by nitrogen allowed for the temperature control of the detonation products. In our previous work, it was shown that the highest coating density and the highest powder deposition efficiency are achieved when spraying of the mixtures is performed in the “hot” spraying mode [11]. In this mode, the particle temperature is higher than the melting point of the metals contained in the mixture [11]. In the present work, the experiments were also performed in the “hot” mode. The spraying parameters were only slightly adjusted. Calculations of the particle in-flight temperatures and velocities performed using the LIH code [14,15] showed that the particles left the barrel in the molten state (Figure 5).

In order to promote the formation of solid solutions, post-treatment was applied to the coatings in the form of laser re-melting. It is important to note that laser re-melting of the coating can be performed with or without partial substrate melting. In the case of partial substrate melting, the coating composition is affected by the procedure due to mixing of the alloy with the material of the substrate. In the present work, in order to avoid changes in the coating composition, only the top surface of the coating was subjected to melting. The laser re-melting procedure was carried out using a fiber laser supplied by IPG Photonics, Ltd. (Oxford, MA, USA) and a YC 52 laser head supplied by Precitec Group (Gaggenau, Germany). A parallel line strategy was used. The parameters of the laser treatment procedure are given in Table 4. These parameters were determined in a preliminary study. In that study, the laser speed and power were varied in order to find the values allowing full re-melting of the top zone of the coating with a depth of the molten pool between 200 µm and 400 µm. 

The X-ray diffraction (XRD) patterns of the as-sprayed and laser-treated coatings were recorded using a D8 ADVANCE diffractometer (Bruker AXS, Karlsruhe, Germany) with Cu Kα radiation. 

The morphology of the powders and microstructure of the coatings were studied by scanning electron microscopy (SEM) using a JEOL JSM7001F microscope (Tokyo, Japan) and a Carl Zeiss Merlin microscope (Oberkochen, Germany) equipped with energy-dispersive spectroscopy (EDS) units. 

## 3. Results

### 3.1. As-Sprayed Precursor Composite Coatings

The composite coatings were successfully deposited from all three selected powder mixtures. The microscopy analysis of the samples did not reveal any cracks or macrodefects in the coatings. No evidence of the coating delaminating from the substrate was found. However, in the composite coatings, some residual porosity was detected.

**Figure 6:**
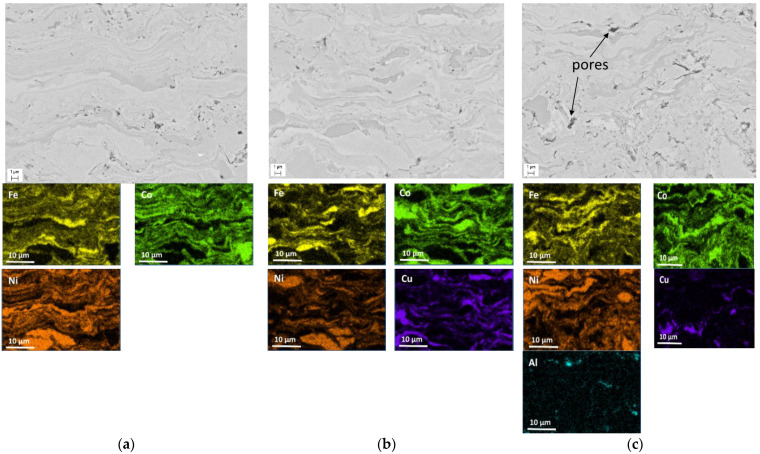
Microstructure (SEM images) and elemental mapping of the as-sprayed coatings: (**a**) Mix 1; (**b**) Mix 2; (**c**) Mix 3.

Figure 6 presents the SEM images of the microstructure and elemental mapping results of the as-sprayed coatings. It is seen that the coatings possess a lamella structure. The particles of the starting powders are distinguishable in the microstructure. The lamellae are uniformly distributed in the coating volume forming a uniform composite structure. At the same time, the evidence of mixing of the metals at the lamella interfaces is observed in the high-magnification images, which confirms the fact that the coatings formed through the deposition of molten particles. At the same time, due to a very high cooling rate during the deposition, the diffusion distances were very short: mixing occurred within the interfacial layers only. 

The results of the EDS analysis of the as-sprayed coatings are presented in Table 5. The measurements were performed in the center of the coating cross-section in five different zones with dimensions 0.25 mm by 0.25 mm. It is seen that the coating composition is different from that of the starting powder mixtures (Table 3). In particular, the concentration of iron in all three coatings is lower than in the starting mixtures. This difference can be explained by a lower deposition efficiency of the iron powder in comparison with Ni, Co, Cu and Al powders. As shown in Figure 5, the applied spraying parameters allowed heating of nickel, copper, cobalt and aluminum to temperatures significantly higher than the melting temperature of the corresponding metal, whereas the iron particle left the barrel at a temperature only slightly above the melting point. As a result, a fraction of the iron particles could cool down in the gas jet after the barrel exit and impact the substrate in the solid state. A similar conclusion was drawn in our previous work [11].

A change in the chemical composition of the coating can affect the thermodynamic and topological parameters of the system, as discussed above. In order to verify whether the parameters in the obtained precursor coatings are still favorable for the formation of HEA, the mixing enthalpy, entropy and atomic size mismatch were re-calculated (Table 5). It can be seen that the only coating meeting the requirements defined by Guo and Liu [13] for the formation of a single-phase solid solution is the one obtained from Mix 3. Nevertheless, laser re-melting was applied to all three coatings. 

### 3.2. Laser Melting of Precursor Coating

**Figure 7:**
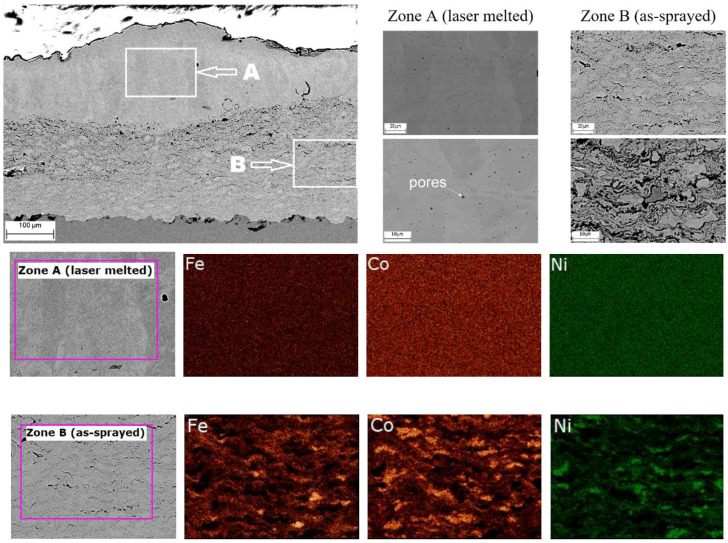
Microstructure (SEM images) and elements mapping of Mix 1 coating after laser re-melting.

A visual inspection of the laser-treated samples did not reveal any signs of coating delaminating from the substrate. No cracks were detected on the coating surface. The microstructure and elemental mapping results of the laser-treated coating obtained from Mix 1 is shown in Figure 7. The boundary between the re-melted zone and the as-sprayed zone is clearly visible. The lamellae of iron, cobalt and nickel in the zone of the as-sprayed material kept their initial shape. In contrast, complete mixing occurred in the melt pool formed during the laser treatment, as evidenced by the microstructure of the re-melted material. Some spherical pores were formed in the re-melted material. The formation of such pores can be attributed to the coalescence of smaller pores initially present in the coating before the laser treatment [16,17]. The EDS maps indicate that the distributions of elements in the re-melted and the as-sprayed zones are different. In particular, a uniform element distribution was observed in the re-melted zone, whereas, in the zones not affected by the laser treatment, lamellae of iron, nickel and cobalt particles could be distinguished. 

**Figure 8:**
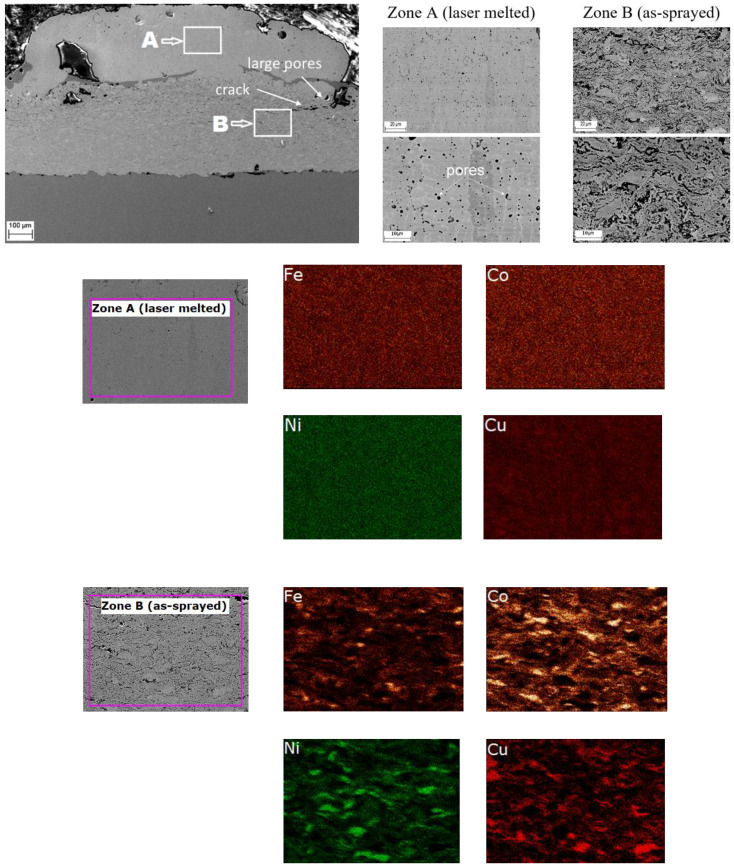
Microstructure (SEM images) and elements mapping of Mix 2 coating after laser re-melting.

The microstructure and element mapping results of the laser-treated coating obtained from Mix 2 (Figure 8) revealed similar phenomena. However, the porosity of this coating in the re-melted zone is higher than that in the re-melted zone of Mix 1 coating. Furthermore, cracks and large pores were found in the transition zone between the re-melted and as-sprayed layers. The formation of these defects can be attributed to partial melting of copper in the transition zone, which affected the coating integrity. Nevertheless, the element distribution in the re-melted zone was uniform such that a homogeneous structure formed. 

**Figure 9:**
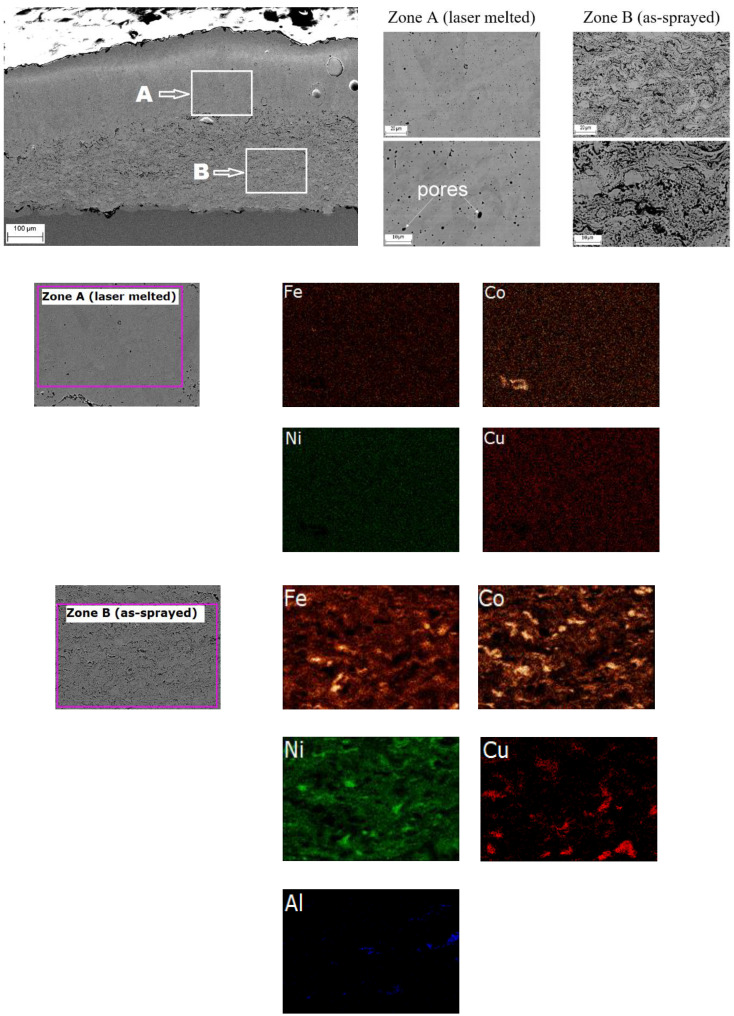
Microstructure (SEM images) and elements mapping of Mix 3 coating after laser re-melting.

Figure 9 shows the microstructure and element mapping results of the laser-treated coating obtained from Mix 3. The element mapping performed on Mix 3 coating revealed the absence of Al in the re-melted zone. Despite its low percentage (~1% at.), aluminum was detected in the zone of the as-sprayed material. Presumably, due to a high temperature of the molten pool during the laser treatment, intensive evaporation of aluminum occurred, which affected the coating composition. 

**Figure 10:**
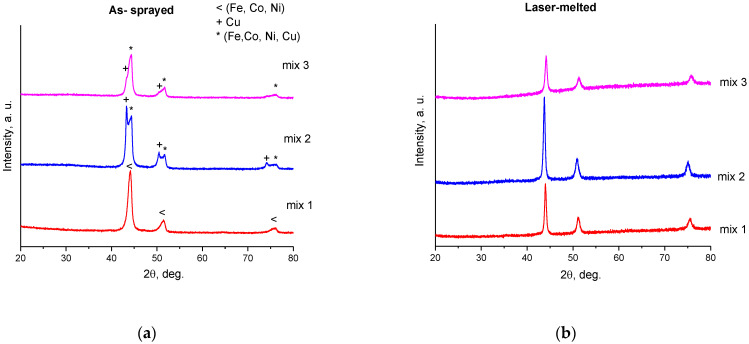
X-ray diffraction patterns of the as-sprayed coatings (**a**) and re-melted layers (**b**). Reflections in patterns shown in (**b**) belong to solid solutions of a face-centered cubic structure. The elemental compositions of the solid solutions are different, as discussed in the text.

The XRD patterns of the as-sprayed and re-melted coatings are presented in Figure 10. In the pattern of the as-sprayed Mix 1 coating (Figure 10a), asymmetrical peaks are observed, indicating the beginning of the formation of solid solutions between the metals. In detonation spraying, the lifetime of the metal particles in the molten state upon the impact is of the order of 100 μs [18]. Therefore, the diffusion of the elements in the as-sprayed coatings occurs only near the interfaces between the solidified particles. In the XRD patterns of the as-sprayed Mix 2 and Mix 3 coatings, peaks of metallic copper are detected. After laser re-melting, the formation of single-phase solid solutions with face-centered cubic (FCC) structure was observed (Figure 10b) for all three compositions. During laser melting, the lifetime of the molten pool is of the order of ~0.1 s, which is sufficient for uniform mixing of the metals in the molten pool. 

## 4. Discussion

The experimental results obtained in the present study demonstrated the feasibility of combining detonation spraying with laser re-melting for the deposition of HEA coatings. In order to further develop this hybrid approach, several critical points should be addressed. 

In the present work, the precursor coatings were deposited by detonation spraying of powder mixtures of pure (unalloyed) metals. It is clear that the prediction of the final coating composition is a difficult task. The deposition efficiency of the mixture components during detonation spraying should be given particular attention. As shown in [11], the efficiency with which particles attach to the surface, depends not only on the detonation spraying parameters, but also on the features of the interaction of the particles of different components with each other during spraying. For example, the deposition efficiencies of iron during detonation spraying of Mix 1, Mix 2 and Mix 3 were different under the same spraying parameters. This indicates that the deposition efficiency of a component depends on the nature of other components of the mixture. In the case of deposition of precursor coatings for the further synthesis of HEA, a small deviation in the coating composition from the target one can significantly change the enthalpy and entropy of mixing and influence the properties of the coatings after re-melting. This issue can be resolved by using agglomerated powders instead of powder blends. 

In the experiments described above, only the top layer of the coatings with a thickness of 0.2–0.5 mm was re-melted in order to avoid the uptake of the substrate material by the alloy. It is clear that this thickness should be optimized, and the influence of the rapid temperature increase on the coating adhesion should be evaluated. The analysis of the mechanical properties of the laser-treated coatings was beyond the scope of the present work. However, those should be studied in the future. 

**Figure 11:**
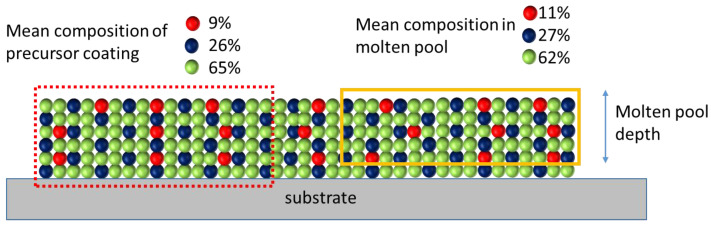
Influence of the size or the molten pool forming during the laser treatment on the final composition of the re-melted layer.

Finally, the possible influence of the size of the molten pool on the deviations of the composition of the re-melted coating from the targeted one should be taken into account. As mentioned above, mixing (alloying between the metals) is the most important factor in the formation of the alloy with the target composition. Figure 11 shows an undesirable situation when the formation of a molten pool influences the composition of the resultant coating; the pool and the remaining (solid) layer may differ in composition. This issue is especially important in the case of re-melting of a precursor coating containing 6–7 elements with the concentration(s) of one or two elements significantly lower than those of other components. This issue can be addressed by decreasing the size of the metal particles in the precursor coatings. In this manner, the compositional uniformity of the coating can be improved. Sintered feedstock powders consisting of agglomerates of metal grains appear to be a viable alternative to powder blends. 

## 5. Conclusions

The possibility of the formation of HEA coatings using a two-stage approach based on the successive application of detonation spraying and laser melting has been shown. The detonation coatings deposited at the first stage had a uniform and dense structure, but the elemental composition of the coatings differed from that of the initial powder mixture due to the differences in the deposition efficiencies of the powders of the components. During the laser treatment applied at the second stage, all components of the precursor coatings melted, which enabled mixing of the metals and alloy formation. A uniform re-solidified layer had a distinct boundary with the as-sprayed zone. The XRD phase analysis indicated the formation of alloys with an FCC solid solution structure in the re-solidified layers. This work allowed validating the two-stage approach to forming HEA coatings. The major drawback of the proposed approach lies in the difficulty of depositing coatings of the target elemental composition when starting from unalloyed mixtures of the metallic components.

## Figures and Tables

**Table 1:** Particle size distribution parameters of the powders used in the experiments.

Powder	D10, µm	D50, µm	D90, µm
Fe	3	12	24
Co	5	11	22
Ni	7	13	26
Cu	6	16	25
Al	5	17	23

**Table 2:** Chemical mixing enthalpies of the atomic pairs between the elements (kJ/mol).

	Ni	Fe	Cu	Al	Co
**Ni**	0	−2	4	−22	0
**Fe**	−2	0	13	−11	−1
**Cu**	4	13	0	−1	6
**Al**	−22	−11	−1	0	−19
**Co**	0	−1	6	−19	0

**Table 3:** Elemental composition of the mixtures used for the precursor coating deposition.

Parameter	Mix 1	Mix 2	Mix 3
Fe, at.%	33	25	30
Co, at.%	33	25	30
Ni, at.%	34	25	30
Cu, at.%	0	25	8
Al, at.%	0	0	2
*ΔS_mix_*, kJ/mol	9.13	11.50	11.33
*ΔH_mix_*, kJ/mol	−2.61	9.99	−0.25
δ, %	1.05	1.14	2.18

**Table 4:** Parameters of the laser melting procedure of the coatings.

Equipment	Power	Spot Diameter	Speed	Scan Strategy	Line Overlapping
IPG + Precitec YC 52	2 kw	3 mm	50 mm/s	Parallel lines	0.5 mm

**Table 5:** Elemental composition of the precursor composite coatings deposited by detonation spraying.

Parameter	Coating Mix 1	Coating Mix 2	Coating Mix 3
Fe, at.%	29.4 ± 1.0	19.1 ± 1.0	25.7 ± 1.0
Co, at.%	34.5 ± 1.0	29.6 ± 1.0	29.5 ± 1.0
Ni, at.%	36.1 ± 1.0	26.6 ± 1.0	33.0 ± 1.0
Cu, at.%	0	24.7 ± 1.0	10.4 ± 0.5
Al, at.%	0	0	1.4 ± 0.1
*ΔS_mix_*, kJ/mol	9.1	11.42	11.39
*ΔH_mix_*, kJ/mol	−2.51	9.25	1.61
*δ*, %	0.32	1.11	1.9

## Data Availability

Not applicable.
